# Motivational communication skills to improve motivation and adherence in cardiovascular disease prevention: A narrative review

**DOI:** 10.1002/clc.24128

**Published:** 2023-09-07

**Authors:** Linqi Xu, Wim Pinxten, Frank Vandereyt, Maarten Falter, Martijn Scherrenberg, Sevda Ece Kizilkilic, Hanne Van Erum, Paul Dendale, Hanne Kindermans

**Affiliations:** ^1^ UHasselt Faculty of Medicine and Life Sciences Diepenbeek Belgium; ^2^ Heart Centre Hasselt Jessa Hospital Hasselt Belgium; ^3^ School of Nursing Jilin University Changchun China; ^4^ Department of Cardiology, KULeuven Faculty of Medicine Leuven Belgium; ^5^ Faculty of Medicine and Health Sciences Antwerp University Antwerp Belgium

**Keywords:** cardiovascular disease prevention, lifestyle behavior change, motivational communication

## Abstract

Lifestyle optimization is one of the most essential components of cardiovascular disease prevention. Motivational counseling provided by health care professionals could promote lifestyle modification. The purpose of the review is to identify possible evidence‐based psychological principles that may be applicable to motivational counseling in the prevention of cardiovascular disease. These motivational communication skills promote behavioral change, improved motivation and adherence to cardiovascular disease prevention. A personal collection of the relevant publications. The review identified and summarized the previous evidence of implementation intentions, mental contrasting, placebo effect and nocebo effects and identity‐based regulations in behavior change interventions and proposed their potential application in cardiovascular disease prevention. However, it is challenging to provide real support in sustainable CVD‐risk reduction and encourage patients to implement lifestyle changes, while avoiding being unnecessarily judgmental, disrespectful of autonomy, or engaging patients in burdensome efforts that have little or no effect on the long run. Motivational communication skills have a great potential for effectuating sustainable lifestyle changes that reduce CVD‐related risks, but it is also surrounded by ethical issues that should be appropriately addressed in practice. It is key to realize that motivational communication is nothing like an algorithm that is likely to bring about sustainable lifestyle change, but a battery of interventions that requires specific expertise and long term joint efforts of patients and their team of caregivers.

## Graphical abstract

Motivational communication for cardiovascular disease prevention. The image shows five components of the communication approach used in motivational counseling around cardiovascular disease prevention.

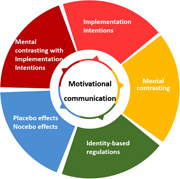

## 1 INTRODUCTION

Sustainable lifestyle changes are essential for many patients to reduce cardiovascular disease (CVD) risk.^1^ The European society of cardiology (ESC) guidelines on CVD prevention in clinical practice have focused on behavior change by highlighting risk‐reducing lifestyle changes, including smoking cessation, physical activity, and a healthy diet.^2^ The implementation of interventions to facilitate lifestyle changes should be promoted by health care professionals. Trying to accomplish this implementation is often complicated and requires much time and effort. Multiple behavior change strategies have been undertaken to increase patients' motivation and adherence. Some communication approaches are also recommended to enhance motivation and adherence.^3^ One of the most popular of these approaches, motivational interviewing (MI) is a useful communication strategy for enhancing motivation and promoting lifestyle change.^2^

MI is directed and person‐centered counseling that aims to change individuals' perceptions of and increase control over their unhealthy behavior. It is based on self‐determination theory and includes communication techniques such as open‐ended questions, affirmation, reflective listening, summarizing, informing, and advising.^4^ The effectiveness of MI as a strategy for eliciting a person's intrinsic motivation to change behavior appears to be strongly supported, according to previously published systematic reviews; however, the findings are constrained by the paucity of primary studies in cardiovascular health settings.^5^ Another systematic review showed that MI is an effective strategy to impact self‐care behaviors, hospital readmission prevention, and quality of life in heart failure patients.^6^ MI is suggested to encourage and engage patients to improve medication adherence.^7^, ^8^ An essential piece of information about the efficiency of MI on primary and secondary prevention of CVD risk factors can be found in one systematic review and meta‐analysis.^9^ The authors concluded that, compared to standard care, MI could positively impact efforts to kick the habit of smoking tobacco and improve psychological variables like depression and quality of life. The authors advised that additional research was needed better to understand the ideal structure and delivery for MI interventions because the results for other outcomes were inconclusive. A recent systematic review addressed the effects of MI on smoking cessation and reported that it is difficult to know whether MI helps people to quit smoking or not because of the few studies and low quality of evidence.^10^ A review by Mifsud^11^ focused on the effectiveness of MI as an intervention to promote risk factor modification in primary prevention. However, no statistical difference between groups in smoking status and physical activity was reported; a random effects analysis from four studies reported standardized mean difference in weight with high statistical heterogeneity. The author suggested that MI delivered by a nurse with expertise, combining MI communication skills with existing education tools, may further support the modification of cardiovascular risk. MI has no exclusivity on motivational communication, and there is a battery of related techniques that could be used complementary or as an alternative.

In this narrative review, we tried to identify possible evidence‐based psychological concepts that could be applied to motivational counseling in CVD prevention and may be helpful in improving motivational communication skills.

## 2 COMMUNICATION APPROACHES USED IN MOTIVATIONAL COUNSELING

An overview of the discussed concepts is depicted in Table 1. An example of communication is shown in the Supporting Information: Appendix 1.

**Table 1:** Motivational communication techniques with definition.

Key communication techniques	Definition
Implementation intentions	Implementation intentions is a goal‐setting technique in which an individual commits to perform a particular behavior when a specific context arises
Mental contrasting	Mental contrasting is a self‐regulation imagery strategy that involves imagining a desired future and mentally contrasting it with the present reality
Mental contrasting with implementation intentions	Combine mental contrasting with implementation intentions
Placebo effect and Nocebo effects	Placebo effect is a psycho‐biological response that occurs in response to a therapy assumed to be beneficial; Nocebo effect is a psycho‐biological response to a purported harmful treatment
Identity‐based regulations	Self‐identity refers to stable and prominent aspects of one's self‐perception

### 2.1 Implementation intentions

Implementation intentions are a goal‐setting technique in which an individual commits to perform a particular behavior when a specific context arises. They supplement goal attainment by specifying an exact future situation and a planned response—“If I encounter situation X, then I will respond with action Y.”^12^ To form an implementation intention, the person must (1) identify a response that will promote goal attainment and (2) anticipate a suitable occasion to initiate that response.^11^

Implementation intentions have been used in various settings to help patients achieve their health goals (e.g., improving physical activity, keeping a healthy diet, quitting smoking) and have a medium‐to‐large average effect on goal attainment.^12^ A systematic review reported that the application of the theory of implementation intentions promoted physical activity behavior among adults in comparison with others that did not use this reinforcement.^13^ Implementation intentions are also proven to be effective both in promoting healthy diets and discouraging unhealthy diets. A study showed that implementation intentions are an efficient intervention that can be used by health education programs to reduce fat intake.^14^ However, a systematic review concluded that sometimes implementation intentions are more effective in promoting healthy diets than diminishing unhealthy diets, although some studies promoting healthy diets effect sizes may have been inflated due to less than optimal control conditions.^15^ Brief, accessible, and easily scalable implementation intentions interventions to prevent smoking are desirable for antismoking efforts.^16^ Implementation intentions as a single intervention were effective for smoking cessation and as part of multicomponent interventions.^17^ Additionally, forming implementation intentions are also proven to be a helpful strategy for helping people with mental health problems to achieve various goals and might be usefully integrated into existing treatment approaches.^18^ For example, systematically taking 10 min of explicit pondering about daily things everyday after dinner can be a good alternative for constant and exhausting worrying and rumination. That shows that implementation intentions have the potential to be applied to cardiovascular disease prevention (e.g., smoking cessation, physical activity, stress) to change patients' behavior.

Implementation intentions could be incorporated into motivational counseling in the context of cardiac rehabilitation to assist patients in developing a practical strategy for making changes and in actualizing those strategies. For example, implementation intentions are a good fit for quitting smoking because smokers frequently identify particular “triggers” of their cravings for cigarettes (e.g., experiencing stress at work).^16^ The implementation intention that “if I experience stress at work, then I will take a walk instead of having a cigarette,” which can serve as the contextual cues for the intended action to prevent smoking, could be set by a person who wants to quit smoking and who is hoping to do so shortly. In this method, the individual might be prepared to effectively manage the encountered trigger through the utilization of implementation intentions. Imagine for a moment that a patient has to engage in a tremendous amount of physical activity. He may formulate an implementation intention such as the following to assist him in reaching this objective: “If I finish my dinner by 8:00 pm, then I will walk around the park for thirty min.” When compared to simply having goal intentions, having implementation intentions also significantly and consistently boosts the likelihood that a person will achieve their goals.^19^ When it comes to facilitating behavior change, the clinicians should interact with the patient to set clear implementation goals during motivational therapy. These goals should include the “when, where, and how” of the implementation.

### 2.2 Mental contrasting

Mental contrasting is a self‐regulation imagery technique to initiate and sustain behavior modification. Mentally contrasting a desired future with encroaching reality can activate expectations of success: when expectations of success are high, individuals commit entirely to and pursue their goals; when expectations of success are low, individuals postpone or quit the pursuit of their desires.^20^, ^21^ Mental contrasting triggers active goal pursuit via three steps: (1) defining an important wish; (2) identifying the best outcome of wish fulfillment and fantasizing about it (defined as free‐flowing thoughts and images describing the best possible outcome); and (3) identifying and then imagining an obstacle in the present reality that prevents the desired future from occurring.^20^, ^21^ Mental contrasting shows promise as a brief behavior change method with a substantial small to moderate effect on improving health behavior in the short term, and the advantages appear to be long‐lasting.^22^ A series of intervention studies showed that mental contrasting could improve participants' academic performance, dietary behavior, and physical activities.^21^

Mental contrasting could transform positive fantasy into binding goal commitment, followed by goal striving. In communicating with patients, try reinforcing the benefits of healthy behaviors and letting patients imagine a positive future. One possible way is to give them positive suggestions which enhance the effectiveness and increase positive outcome expectations regarding cardiac rehabilitation.^23^ Although a study has shown that this approach does not enhance objective outcomes, it can influence subjective feelings.^23^ However, thinking only about positive future outcomes decreases goal‐relevant efforts. It is also necessary to think about obstacles that impede the realization of wishes.^23^ Understanding perceived CVD risk and realizing negative results of CVD risks create a psychological contrast in the patient's mind, where the desire for benefits and the contrast with reality will motivate patients to take action. Studies showed that 40% of patients underestimate their CVD risk.^24^ Patients' perceived absolute CVD risk was associated with willingness to take prevention therapy.^25^ It is necessary to combine motivational counseling with risk education and use existing CVD risk tools to help assess CVD risks. However, another study evaluated calculated and perceived CVD risk among 4187 patients and found that most patients overestimated their 10‐year cardiovascular risk.^26^ In this case, providing accurate absolute risk assessments to patients without proper context may paradoxically decrease many patients' perceived risk of CVD, thereby disincentivizing the initiation of CVD risk reduction therapy. The evidence is thus inconclusive, tailored solutions need to be proposed for different individuals.

### 2.3 Mental contrasting with implementation intentions

Mental contrasting is a goal‐setting approach that can convert positive fantasy into binding goal commitment, followed by goal striving; implementation intention is a goal‐implementation strategy that supplements goal intention and motivates action. The efficiency of implementation intentions has been drastically enhanced by combining them with mental contrasting. Due to the expanding volume of studies on this dual strategy, this combination deserves special note.^27^ According to laboratory and field research, combining mental contrasting with implementation intentions (MCII) is more effective in goal pursuit than mental contrasting or implementation intentions alone.^28^, ^29^ A meta‐analysis found that MCII is a straightforward and effective technique for goal attainment with a small to moderate effect; however, the actual effect sizes may be smaller due to publication bias.^30^ Nonetheless, Cross and Sheffield found that MCII had a small‐to‐medium effect on health‐related behavior, comparable to a mental intervention's effect size.^22^

People are not always able to properly transform their high goal commitment into goal‐directed behavior after forming it through mental contrasting. For instance, individuals may forget to act; confront challenging impediments such as urges, intense emotions, or ingrained habits; or be unaware of prospective action signs.^21^ Implementation intentions are additional techniques for overcoming such hurdles because they reinforce the relationship between difficulties and goal‐relevant instrumental behaviors to overcome obstacles, which is generated by mental contrasting.^31^ MCII enables patients to define their desired future independently, identify obstacles, and formulate an “if obstacle, then I will behavior” plan. It contrasts with most implementation intentions studies, in which researchers provide compelling content to be inserted in If–then plans. For example, “If I do not have time or forget to run in the morning, I will do that in the evening by 8 o'clock.” That would serve as a reminder for patients to engage in healthier behaviors. Another example is when patients resist the temptation to buy unhealthy snacks (that threaten the weight goal or desired cholesterol reduction). Patients can opt to choose from a grocery list while they are shopping. In this case, patients could not only identify and imagine an obstacle in the present reality that prevents the desired future from occurring but also especially eliciting coping strategies to handle the obstacle. The method of planning coping responses can be an interesting motivational skill. Participants may generate disappointing outcomes, high obstacles, or ineffective plans for overcoming obstacles, which may reduce the effect of the MCII interventions, even though autonomous use of MCII significantly increases the scale of application in everyday life.

### 2.4 Placebo effect and nocebo effects

The placebo effect is a psychobiological response that occurs in response to a therapy assumed to be beneficial.^32^, ^33^ The hypothesis that a person's expectations play a role in the placebo effect is widely evidenced by research. The placebo effect operates in three possible pathways: expectations, patient–physician relationship, and classical conditioning. If a person takes a tablet with the expectation that it will have a particular impact, then it is feasible that the individual's body chemistry will cause effects comparable to those the pill might have caused. The concept that the placebo effect contributes in some way to the psychological benefits of exercise and physical activity is supported by studies.^32^

An experiment was carried out by Crum and Langer^34^ in which 84 female room attendants working in seven different hotels were measured on physiological health factors that are affected by exercise. Those who were placed in the informed condition had the information relayed to them that the task they do (cleaning hotel rooms) is beneficial exercise and complies with the guidelines made by the Surgeon General for leading an active lifestyle. There were presented some examples of how their work could be considered exercise. The information in question was withheld from the participants in the control group. However, there was no difference in the participants' actual activity 4 weeks following the intervention, those in the informed group perceived that they were exercising substantially more than they had previously. As a result, the placebo group showed a reduction in weight, blood pressure, body fat, waist‐to‐hip ratio, and body mass index compared to the control group. That makes it abundantly evident that people's perceptions of the benefits of physical activity directly correlate to their health. When it comes to preventing CVD, increasing adherence to physical activity can be challenging. We can inform patients about the physical activities they are already doing around the house, picking up and dropping off children, and other daily physical activities; alternatively, we can advise them about feasible ways to mix exercise with their everyday activities.

The nocebo effect is the opposite, a psychobiological response to a purported harmful treatment.^33^ Physical sensations like discomfort and itching are known to be affected by nocebo effects or adverse therapeutic effects caused by patients' expectations. Myers et al.^35^ reported that discussing potential side effects resulted in further withdrawals from the research. A greater understanding of reducing nocebo responses may eventually result in enhanced adherence to therapeutic regimens.^36^ It is challenging to inform patients about the potential adverse effects of pharmaceutical treatments or the measures they should take when engaging in physical activity to reduce the likelihood of experiencing a placebo effect when it comes to preventing cardiovascular disease. Historically, clinicians have paternalistically controlled the disclosure of information to patients,^37^ but both the law and medical ethics have endorsed informed consent and strongly restricted the “therapeutic prerogative” to withhold information.^38^ Therefore, clinicians face the ethical dilemma of communicating so that nocebo responses can be minimized in clinical practice consistent with informed consent. Colloca^39^ reported several communication skills to minimize nocebo effects. For instance, a given side effect can be mentioned merely as a possibility, using a positive framing to report side effects, and using the authorized concealment technique. For instance, telling patients that research indicates that discussing all possible adverse effects will increase their likelihood of experiencing them, and then asking if they want to be told and how much information they want. This context of “level of information” is an essential motivational aspect often seen in the clinical practice of cardiac rehabilitation where for example, one patient indicates to be anxiously overpowered by the details his cardiologist gave him about his valve operation while the other patient claims that detailed medical information helps him in mastering the process of acceptance. Probing patients desired levels of information (in general and before cardiac interventions or medicine prescriptions) can be helpful.

Specific motivational aid in placebo and nocebo effects can be the technique of Belief selection, using messages designed to strengthen positive beliefs, weaken negative beliefs, and eventually introduce new beliefs.

### 2.5 Identity‐based regulations

Self‐identity refers to stable and prominent aspects of one's self‐perception (e.g., “I think of myself as a green consumer”).^40^ Several studies have linked the concept of self‐identity with the theory of planned behavior (TPB) and reported that identity independently contributes to predicting behavioral intentions.^41^, ^42^ Identities provide a behavior standard,^43^ allowing individuals to assess the congruence between the meanings of their behaviors and their identities. This appraisal may elicit negative or positive feelings, and individuals may alter their activities if they are inconsistent with identity expectations.^44^

Evidence showed that identity could predict behavior. Theodorakis^45^ demonstrated that role‐identity about physical activity explains both intent and conduct. Strachan and Brawley^46^ revealed in a study on healthy‐eater identity and self‐efficacy as predictors of healthy eating behavior that these variables enhanced the variance in healthy eating behavior that could be explained. Brouwer and Mosack^47^ demonstrated that identity as a healthy eater was a significant predictor of healthy eating intentions beyond the TPB components and a significant predictor of overall healthy eating behaviors, fruit and low‐fat dairy intake beyond intentions and perceived behavior control. A new longitudinal study examined the identity processes of smokers who wish to quit and found that those who wrote about their future as nonsmokers were more effective at quitting.^48^ By discussing the benefits and drawbacks of being a smoker and nonsmoker, it is possible to persuade the patient to become a nonsmoker and commence identity transformation.

When it comes to motivating communication to prevent cardiovascular disease, it will be very beneficial to adopt methods that will assist patients in acquiring a healthy identity (e.g., being an active, fit, nonsmoking person). For example, the interaction between improving motivation, adherence, and the identity of being a heart patient in terms of a successful adaptation process can contribute to decoupling patients' self‐esteem from compromised health: “I have a heart condition, but now I choose consciously for a (cardiac) healthy lifestyle. Moreover, this gives me much more quality of life now than before my hospitalization.” In particular, when mental contrasting with implementation intentions contributes to a healthy identity, motivation and communication skills can make the difference.

## 3 DISCUSSION

Lifestyle changes have an important potential to reduce CVD risks, but pose important challenges for both patients and professional caregivers. For patients, it is difficult to change behaviors in a sustainable way. Many efforts to do so fail, which may induce feelings of guilt or hopelessness. For professionals, it is challenging provide real support in sustainable CVD‐risk reduction and encourage patients to implement lifestyle changes, while avoiding being unnecessarily judgmental, disrespectful of autonomy, or engaging patients in burdensome efforts that have little or no effect on the long run. This raises ethical concerns, which can be discussed in reference to the four major principles of biomedical ethics: autonomy, beneficence, nonmaleficence, and justice.^49^

First, in terms of autonomy, motivational communication offers great advantages. It is patient‐centered, respectful of autonomy and antipaternalistic in the sense that patients can self‐define their own goals and the way in which they want to reach them. In addition, it builds on the willingness to change behaviors that is already present in the patient. Nonetheless, autonomy also has a downside. Starting from self‐defined goals and self‐chosen approaches to realize these goals, patients are put in charge of making choices, but may equally end up in making poor or bad choices. This may result in senseless efforts to reduce CVD risks and might even keep patients away from other goals and approaches that are much more promising. Adequate counseling is therefore crucial, be it in a way that remains respectful of autonomy. Practitioners should therefore be vigilant of being overly directive in their motional communication, while still adequately informing patients about the options of choice they realistically have.

Second, there is the principle of beneficence. Given that motivational communication has a great potential for actual impact while avoiding overreliance on merely informing or instructing patients, it has everything in it to be beneficent. In addition, it does not restrict to curative responses to disease symptoms but encompasses an active commitment to the health of patient, and as such it constitutes an active effort to bring benefit to the patient.

Third, motivational communication should be respectful of the principle of nonmaleficence, which implies to “first do no harm.” While intentional harm to patients is unlikely, there is a risk for unintentional or hidden harms. Initiatives to discuss behavioral change may for example, experienced as intrusive or even oppressive for people who are not open to behavioral change. The willingness, context, and timing should therefore be carefully considered. If the eventual outcome of motivational communication interventions is disappointing, this may provoke normative judgments in both patients and professionals that are for example associate failure with personal weakness and invoke burdensome and undesired feelings of failure, shame, guilt, or discouragement in patients. As there is no guarantee that motivational communication will effectuate the desired effects, strategies to help coping with failure should be build in when implementing motivation communication in clinical practice.

Finally, the use of motivational communication also generates questions related to justice. Vigilance to inappropriate attribution of personal responsibility is required, and the socioeconomic and cultural factors that influence the likelihood of success should be better studied. It is well possible that when taking these factors into account, not everyone has an equal chance to positive outcomes. Taking the risk to blame and guilt or potential adverse effects of persistent failure into account, this can generate important heath inequities.

## 4 CONCLUSION

Summarized, motivational communication has a great potential for effectuating sustainable lifestyle changes that reduce CVD‐related risks, there might be a gaining effect if health care professionals integrate implementation intentions, mental contrasting, placebo effect, nocebo effects, and identity‐based regulations into motivational counseling. But it is also surrounded by ethical issues that should be appropriately addressed in practice. Against this background, it is key to realize that motivational communication is nothing like an algorithm that is likely to bring about sustainable lifestyle change, but a battery of interventions that requires specific expertise and long term joint efforts of patients and their team of caregivers.

## Supporting information

Supporting information.

## Data Availability

Data sharing not applicable—no new data generated. This review article does not present new data, as its primary objective is to critically evaluate and synthesize existing literature. Researchers interested in exploring the data sources referenced in this review are encouraged to consult the original publications cited for further information.
