# Supplementary material for: Motivational communication skills to improve motivation and adherence in cardiovascular disease prevention: A narrative review
Source: Clin Cardiol. 2023 Sep 7;46(12):1474–80. doi: 10.1002/clc.24128 (PMC10716351; doi:10.1002/clc.24128)
Supplement: Supplementary file 1 — Supporting information. [file CLC-46-1474-s001.docx]

# Appendix: An example of communication

Clinical practitioner A: Cardiac rehabilitation and secondary prevention is beneficial in preventing further cardiac events. It is associated with a 20% to 30% reduction in mortality. Just imagine, if you do cardiac rehabilitation from now, you might get benefits of strengthening your heart and body, relieving symptoms of heart problems, such as chest pain, building healthier habits such as getting more physical activity, quitting smoking, and eating a heart-healthy diet, reducing stress, improving your mood... **(Mental contrasting)**

Clinical practitioner A: Would you like to make a heart healthy lifestyle choice from now on? **(Self identity)**

Patient A: I have a heart condition, but now I choose consciously for a cardiac healthy lifestyle. Moreover, this gives me much more quality of life now than before my hospitalization. **(Self identity)**

Clinical practitioner A: OK, that’s start with the physical activity then. What kind of physical activity do you do now?

Patient A: I didn’t do exercise specifically. But I need to ride the bikes to pick up my children 5 days per week... It was around 20 minutes in total.

Clinical practitioner A: Physical activities are not only what you do in the gym or rehabilitation center, but also include what you are already doing around the house, such as picking up and dropping off children, and other daily physical activities... You could also mix exercise with your everyday activities. So the 20-minute cycling is also considered to be a physical activity. **(Placebo effect)**

Clinical practitioner A: In cardiac rehabilitation, you need to do 150-300 minutes of moderate-intensity physical activity or 75-150 minutes of vigorous-intensity physical activity per week **(Mental contrasting).** Now the physical activity you are doing is not enough. Would you like to add some extra physical activity?

Patient A: I would like to try jogging from now on. How long should I do?

Clinical practitioner A: If you already do the cycling, It will be good for you to do 20 minutes per day, 3-5 days per week. When and where do you want to do it?

Patient A: I am not sure yet. Maybe in the morning.

Clinical practitioner A: It would be better if you could set a specific plan for yourself. Would you like to set a physical activity goal for yourself? For example, if you get up by 7:00 AM, then you will jog around the park for thirty minutes. **(Implementation intentions)**

Clinical practitioner A: If you find some obstacles in jogging in the morning, for example, you could not get up by 7:00 AM, you need to do something else like taking care of your children. You could also set a goal like: "If you do not have time or forget to jog in the morning, you will do that in the evening by 8 PM." **(Mental contrasting with Implementation Intentions)**

Patient A: Will jogging have an adverse effect on my heart?

Clinical practitioner A: There are some research indicates that discussing all possible adverse effects of physical activity will increase their likelihood of experiencing them, do you want to be told and how much information you want? **(Minimize nocebo effect)**.
